# Endoscopic Closure of Iatrogenic Ventricular Septal Defect After 2 Previous Aortic Valve Replacements

**DOI:** 10.1016/j.jaccas.2025.104166

**Published:** 2025-07-16

**Authors:** Abdelrahman Abdelbar, Simran Kundan, Andrew Knowles, Joseph Zacharias

**Affiliations:** a Cardiac Surgery Department, Manchester Foundation Trust, Manchester, United Kingdom; b Cardiac Surgery Department, Blackpool Teaching Hospitals, Blackpool, United Kingdom

**Keywords:** aortic valve, acute heart failure, valve replacement, ventricular septal defect

## Abstract

**Background:**

A 79-year-old male patient previously had undergone 2 aortic valve replacements via median sternotomy.

**Case Summary:**

He presented with progressive shortness of breath shortly after the second procedure. This led to the identification of an iatrogenic perimembranous ventricular septal defect on a transthoracic echocardiogram. A heart team discussion concluded that the defect was not amenable for percutaneous closure. An endoscopic minimally invasive surgical closure was performed, and the patient had a quick recovery.

**Discussion:**

To the best of our knowledge, this is the first case of an iatrogenic ventricular septal defect closure via an endoscopic approach. This highlights the feasibility of using this approach in complex redo cardiac conditions to avoid further sternotomies.

**Take-Home Message:**

Heart teams should consider an endoscopic approach with complex cases specifically if there is no interventional option available.

## Graphical Abstract

**Figure:**
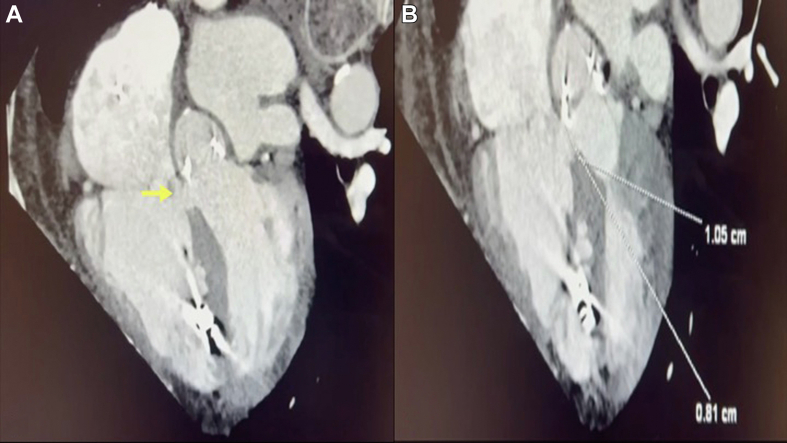

## History of Presentation

A 70-year-old gentleman, with 2 previous tissue aortic valve replacements (AVRs), presented with signs of right heart failure. The first surgery was in 2008 for severe aortic stenosis, and the second surgery was in 2021 for structural degeneration of the bioprosthetic valve. He developed an iatrogenic ventricular septal defect (VSD) after the second AVR. Both the times, the valve was approached via median sternotomy. After the second valve replacement, the patient developed a complete heart block, which required permanent dual chamber pacing. Within 18 months of the procedure, he developed progressive shortness of breath with NYHA functional class III-IV. This required multiple hospital admissions with heart failure and pleural effusion requiring drainage. A transthoracic echocardiogram (TTE) revealed a perimembranous VSD with raised pulmonary artery systolic pressure (PASP).

**Take-Home Message**

• An endoscopic procedure is a versatile approach in the armamentarium of a cardiac surgeon to tackle redo situations, specifically in complex cases with no available percutaneous options.

## Past Medical History

His past medical history included 2 previous AVRs, with the last one being in 2021, and transvenous placement of a permanent pacemaker for complete heart block.

## Differential Diagnosis

The differential diagnoses included structural valve failure including bioprosthetic valve degeneration or other new native valve pathology, patient-prosthesis mismatch, new-onset coronary artery disease, pacemaker-induced cardiomyopathy, or VSD.

## Investigations

An electrocardiogram revealed paroxysmal atrial fibrillation with a fast ventricular rate (the pacemaker was set to dual chamber pacing backup). Postdischarge TTE (after the second AVR) revealed a dilated left ventricle, with normal systolic function. There was flattening of the interventricular septum (IVS) because of high right ventricular (RV) pressures. The RV was mildly dilated with normal function. The aortic valve prosthesis was well seated, with a mean pressure gradient of 10 mm Hg across. Mild-to-moderate mitral regurgitation and mild tricuspid regurgitation (TR) were noted. A left-to-right flow was visualized across the perimembranous region of the IVS. The patient was, however, asymptomatic, so a close follow-up was instigated.

One-year follow-up TTE revealed a dilated right atrium and RV with impaired systolic function. The left ventricle was mildly dilated with borderline systolic function. The aortic prosthesis still appeared well seated, with a mean pressure gradient of 4 mm Hg. Moderate-to-severe TR and mild-to-moderate mitral regurgitation were again noted. An estimated PASP of 81 mm Hg was noted. The left-to-right flow across the IVS had a peak velocity of 4.5 m/s. These findings were confirmed with a transesophageal echocardiogram (Video 1).

**Figure 1:**
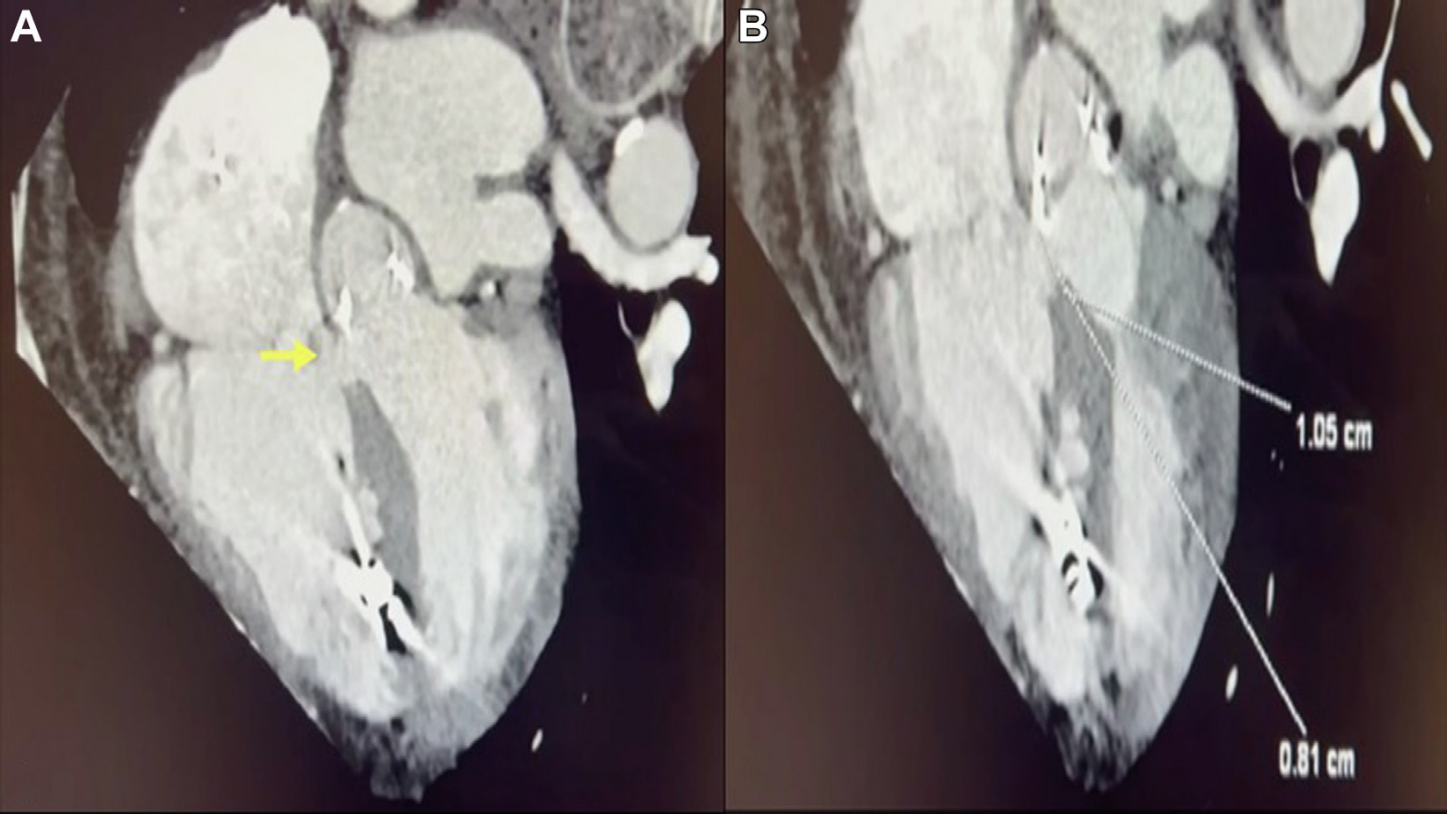
Cardiac CT Scan Cardiac CT showing the ventricular septal defect (A) and the measurements (B). CT = computed tomography.

An electrocardiogram-gated cardiac computed tomography scan confirmed a perimembranous VSD measuring 10 mm with dilated right atrium and RV. It also showed suitable femoral vessels for peripheral cannulation for cardiopulmonary bypass (CPB). The ascending aorta was also found suitable for using endoluminal aortic occlusion (Figure 1).

## Management (Medical/Interventions)

**Figure 2:**
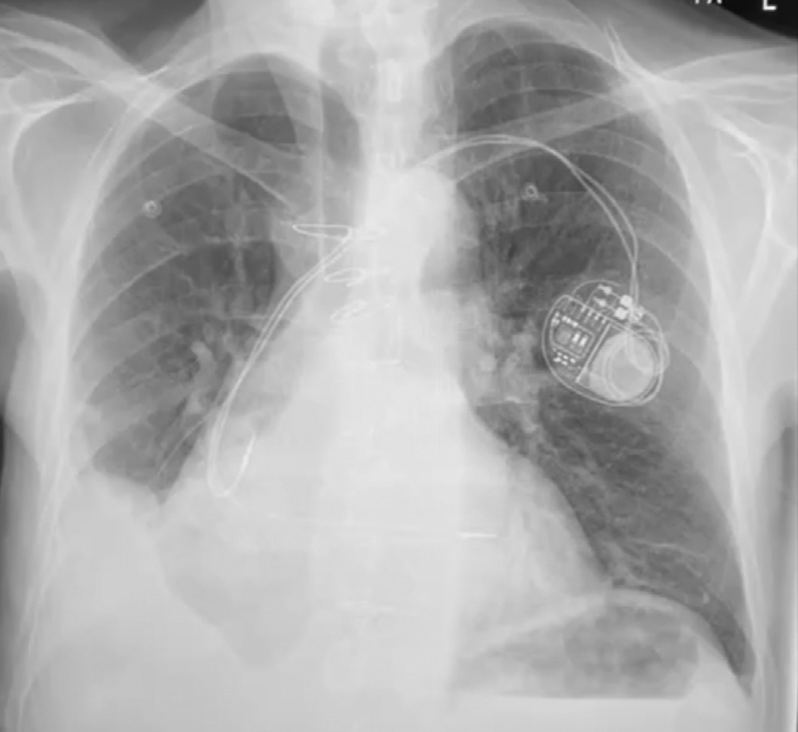
Chest Radiograph Showing Right-Sided Pleural Effusion

The patient was started on medical therapy for heart failure as an outpatient, but as the symptoms progressed to congestive heart failure, he was admitted to our hospital. In addition to aggressive heart failure therapy, a large right-sided pleural effusion was drained (Figure 2). In keeping with the left-to-right shunt with raised PA pressure, the multidisciplinary team decision was to close the VSD. The percutaneous option was deemed not suitable because of the proximity to the prosthetic valve with limited landing rim and because of the proximity to the tricuspid valve, which could cause tricuspid valve malfunction. Because the patient already had 2 sternotomies, we offered him an endoscopic minimal-access VSD closure.

## Procedure

The CPB was established via the right femoral vessels with a 21-F EndoReturn cannula (Edwards Lifesciences) for arterial cannulation and a 23 multistage venous return cannula (Medtronic). An intraluminal balloon (Endoballoon) occlusion device (IntraClude; Edwards Lifesciences) was placed through the EndoReturn cannula and positioned at the ascending aorta ready for inflation.

**Figure 3:**
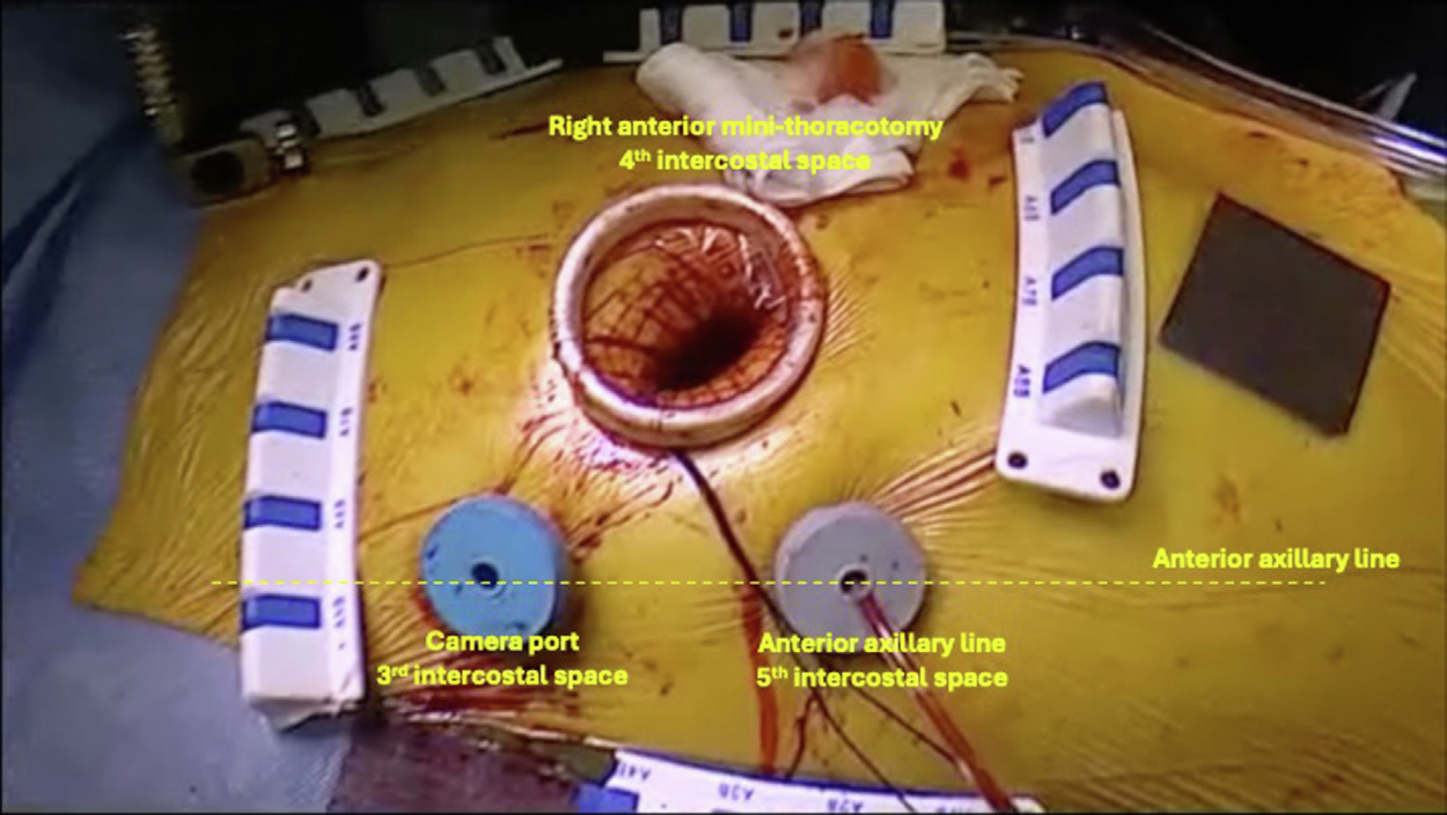
Surgical Setup Showing the Surgical Incision and Positioning of the Ports

**Figure 4:**
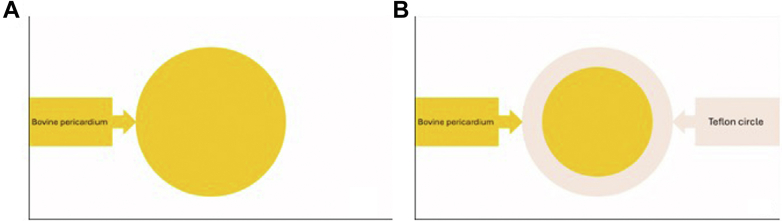
Patch Preparation Patch preparation with a bovine pericardial patch (A) and a Dacron ring (B).

A small (4 cm) anterior minithoracotomy was performed after institution of CPB and deflation of the lungs. Two other ports were placed in the fourth and fifth intercostal spaces at the anterior axillary line for the 3-dimensional endoscope and vent suction (Figure 3). This enabled safe entry to the right pleura and dissection of the pleural adhesions caused by the previous 2 surgeries and the repeated right pleural aspiration for heart failure. The heart was arrested using the IntraClude device when satisfactory exposure of the pericardium covering the right atrium was achieved. The right atrium was opened in one block with the covering pericardium (Video 2). The VSD was located just below the anteroseptal commissure of the tricuspid valve (Video 3), so we had to detach the anterior part of the septal leaflet (Video 4). The VSD was closed using interrupted pledgeted 2-0 Ethibond (Ethicon, Johnson&Johnson) sutures (Video 5) and a pericardial patch reinforced with a Dacron (Macquet) ring at the periphery (Figure 4). The patch was secured with an automated knot fastener (COR-KNOT; LSI Solutions) (Video 6). The anterior part of the septal leaflet was reattached with running 4/0 Prolene (Ethicon, Johnson&Johnson) suture with closure of the anteroseptal commissure to prevent TR (Video 7). After closure of the right atrium, the patient was weaned smoothly from CPB. Intraoperative transesophageal echocardiogram confirmed complete VSD closure and competent tricuspid valve (Video 8).

## Outcome and Follow-Up

The patient was extubated 4 hours after surgery and discharged to the ward the following day. He was fully mobilized on day 4 and discharged by the physiotherapy team. Finally, he was discharged home on day 6 postoperatively.

He had a TTE before discharge and at 6 months. Both showed reduction of both PASP and RV pressure and also confirmed no residual flow across the VSD patch repair (Video 9).

## Discussion

Median sternotomy is the standard of care for cardiac surgery and its complications at present.^1^ Minimally invasive endoscopic cardiac surgery is increasingly being used in centers for first-time heart valve surgery particularly in disorders of the mitral valve.^2^ Endoscopic surgery offers smaller incisions, reduced blood loss, earlier discharge, and better patient satisfaction.^3^ Despite the growing interest in this approach, the use of endoscopic techniques for redo surgery is still limited to expert surgeons and experienced centers.

Presently, patients presenting with complications after sternotomy are increasingly considered for transcatheter solutions because of the higher morbidity and mortality after redo sternotomy.^4^ There has been a rapid increase in case reports of transcatheter solutions to difficult problems, and this has made an impressive difference to the patient outcomes and pathways post intervention.^5^ As in this case, there are limitations to transcatheter options, and in this case, it was the fact that the rim of the VSD was too close to the ring of the prosthetic valve. We discussed this case with multiple interventional cardiologists to see if there was a nonsurgical option available. Subsequent to discussion at 2 separate centers, the consensus was to consider a surgical approach, and this was discussed with the patient. We made the patient aware that we had limited experience with this approach but felt an endoscopic procedure would be better tolerated than a third sternotomy.

In this case report, we would like to highlight the versatility of the endoscopic approach. We used femoral vessels for institution of bypass. We routinely use the Endoballoon for cross-clamping the aorta, and this greatly facilitates the ease of myocardial protection. We have previously published an endoscopic repair of a postinfarct VSD using a very similar approach.^6^ This approach can also be used for first-time VSD repairs in the setting of congenital defects.

We present this case to highlight the complimentary role that endoscopic approaches play in heart team discussions in the future. We believe that in some patients, an endoscopic solution should be considered, and because these techniques are limited to some expert centers at present, discussion with these centers may be in the best interest of patients with complex cardiac conditions.

Most endoscopic procedures are seen as a cosmetically superior option than the traditional sternotomy approach.^7^ In the setting of this patient, the main advantage of the endoscopic approach was the ability to get to the VSD without a prolonged period of risky dissection of the retrosternal cardiac structures. We feel this helps surgeons to perform complex procedures with better visualization. It helps hospitals as the time of the procedure and the time spent in intensive care and the ward is less demanding as seen in this case.

We believe this may be the first case of this nature dealt with by an endoscopic approach but hope it will be used increasingly in the future.

## Conclusions

Iatrogenic VSD is a rare complication of AVR, especially in a redo situation. This case report highlights the importance of diagnosis and early closure to prevent irreversible pulmonary artery hypertension. Although median sternotomy is the standard approach for redo cardiac surgery, an endoscopic approach is a viable option as it simplifies the process of dissection and correction of the pathology.

**Visual Summary:** Timeline of the Case

Timeline	Events
2008 (First procedure)	Aortic valve replacement via median sternotomy with a biological bioprosthesis due to severe aortic stenosis.
2021 (Second procedure)	Tissue aortic valve replacement via redo sternotomy due to structural degeneration of the bioprosthesis.
14 d After the second procedure	Permanent dual-chamber pacemaker fitted due to complete heart block
18 mo After the second procedure	Multiple admissions with heart failure and pleural effusion TTE, TEE, and cardiac CT confirmed an iatrogenic VSD
Hospital admission	Heart team discussion: not suitable for percutaneous closure Accepted for surgical closure of the VSD
2022 (Third procedure)	Endoscopic surgical closure of the VSD Discharged home on day 6 post-surgery

CT = computed tomography; TEE = transesophageal echocardiogram; TTE = transthoracic echocardiogram; VSD = ventricular septal defect.

## Funding Support and Author Disclosures

Dr Zacharias has received honoraria or speaker fees from Edwards Lifesciences, Medtronic, Abbott, Cambridge Medical Robotics and Intuitive Surgical. All other authors have reported that they have no relationships relevant to the contents of this paper to disclose.

## Appendix

Video 1 Preoperative Transesophageal Echocardiogram Showing the Ventricular Septal Defect and Tricuspid Regurgitation

Video 2 Right Atriotomy in One Block With the Pericardium

Video 3 Locating the Ventricular Septal Defect Just Below the Anterior Part of the Septal Leaflet

Video 4 Detaching the Anterior Part of the Septal Leaflet

Video 5 A Surgical Video Showing Placement of Teflon Pledged Ethibond Sutures on the Ventricular Septal Defect Margin

Video 6 Parachuting and Securing the Repair Patch Using COR-KNOT

Video 7 Reattaching the Septal Leaflet With Commissure Closure

Video 8 Postprocedural Transesophageal Echocardiogram Showing Competent Tricuspid Valve and No Residual Flow Across the Ventricular Septal Defect

Video 9 Six-Month Follow-Up Transthoracic Echocardiogram With No Residual Flow Across the Ventricular Septal Defect

Case Video A Narrated Video Presentation of the Whole Case
